# Sexual risk behaviour among school-going adolescents in Sierra Leone and Liberia: a secondary analysis of the 2017 Global school-based student health surveys

**DOI:** 10.1186/s40834-022-00193-w

**Published:** 2022-12-24

**Authors:** Peter Bai James, Augustus Osborne, Abdulai Jawo Bah, Emmanuel Kamanda Margao, Mohamed Conteh-Barrat

**Affiliations:** 1 National Centre for Naturopathic Medicine, Faculty of Health, Southern Cross University, Lismore, Australia; 2 Faculty of Pharmaceutical Sciences, College of Medicine and Allied Health Sciences, University of Sierra Leone, Freetown, Sierra Leone; 3 Department of Biological Sciences, School of Environmental Sciences, Njala University, Njala Campus, Njala, Sierra Leone; 4 Institute for Global Health and Development, Queen Margaret University Edinburg, Musselburgh, Scotland, UK; 5 St. Joseph’s Catholic Hospital, Monrovia, Liberia

**Keywords:** Adolescents, Sexual risk behaviour, Sierra Leone, Liberia

## Abstract

**Background:**

Sierra Leone and Liberia have experienced civil wars and, recently, Ebola outbreaks that led to profound economic hardship, psychopathologies and family disruptions. These factors are associated with sexual risk behaviours among youths. However, there is very little information on sexual risk behaviour among Sierra Leonean and Liberian school-going adolescents. The present study assessed the prevalence and determinants of sexual risk behaviours among school-going adolescents (10–19 years) in Sierra Leone and Liberia.

**Method:**

We used publicly available nationally representative cross-sectional datasets of the 2017 Sierra Leone and Liberia Global school health survey. The sample consisted of 2798 and 2744 school-going adolescents from Sierra Leone and Liberia, respectively. Complex sample descriptive and regression analysis was used to analyse our data.

**Results:**

The majority of adolescents in the two countries were involved in multiple sexual risk behaviour (80.2%), with a higher prevalence observed in Sierra Leone (85.2%) than in Liberia (75.3%). Liberian adolescents showed lesser odds of indulging in multiple sexual risk behaviours than their Sierra Leonean counterparts (AOR = 0.572; 95%CI: 0.345–0.946). Male, compared to females, were more likely to engage in multiple sexual risk behaviour (AOR = 2.310;95%CI:1.543–3.458), with a similar pattern observed in both countries. Alcohol use was associated with multiple sexual risk behaviour (AOR = 3.064; 95%CI: 2.137–4.392). Also, in Sierra Leone and Liberia, adolescents with one and two or more forms of psychological distress were more likely to have ever had sex than those who did not show any form of psychological distress. Missing class/school was associated with multiple sexual risk behaviour (AOR = 1.655; 95%CI:1.133–2.418). Peer support was only found to be a protective factor against no condom use among Liberian adolescents (AOR = 0.608; 95%CI: 0.435–0.850). Less parental support was only associated with ever had sex among adolescents in Sierra Leone (AOR = 2.027; 95%CI: 1.322–3.107) but not in Liberia (AOR = 1.034; 95%CI: 0.650–1.644).

**Conclusion:**

Our study found a high sexual risk behaviour among school-going adolescents in Sierra Leone and Liberia. Our finding highlights the need to strengthen sexual and reproductive health education in schools and communities that incorporate mental health promotion activities tailored to this group.

**Supplementary Information:**

The online version contains supplementary material available at 10.1186/s40834-022-00193-w.

## Background

Youths in sub-Saharan Africa are prone to risky sexual activities, unwanted pregnancy, and sexual violence [1, 2]. Having unprotected sexual intercourse; having multiple sexual partners over one's lifetime; having intercourse with a casual partner; sexual initiation at a young age; sexual intercourse with commercial sex workers; bartering sex for money, goods, or other favours; engaging in sexual activity while under the influence of alcohol/drugs; and sexualism are all examples of risky sexual behaviours [3].

Adolescence (10–19 years) is characterised by greater autonomy, peer influence, and risk-taking behaviours such as initiation of sex and alcohol/drug usage [1, 4]. Compared to adults, adolescents are more likely to have several sexual relationships, participate in unprotected sexual intercourse, and choose high-risk partners [5]. The study of teenage sexual behaviour is crucial because 60% of youths globally are afflicted with sexually transmitted infections (STIs), including HIV [6].

Earlier studies in Liberia reported a significant prevalence of risky sexual practices among in-school children and young adults aged 12-36 years. Seventy-eight% of school children and young adults were found to be sexually active; 13% of those sexually active had sex for money, while 25% seldom or never used a condom [7]. In the same study, males had many sexual partners, and start sex earlier than females. Another study among Liberian adolescents (age range = 13–19 years) found that 34% of those who were sexually active did so before the age of 15 years. Twenty-one percent of those who were sexually active had several sexual partners, and 26% of sexually active teenagers had never used a condom, 11% had gotten pregnant or helped someone become pregnant one or more times, and 16% had been sexually assaulted [8]. A recent study reported that majority of sexually active Sierra Leonean adolescents aged 15–19 years had condomless sex in their last sexual encounter [9]. A United Nations Population Fund report on the impact of Ebola on adolescent pregnancy in Sierra Leone found that,nearly half had their first pregnancy during the Ebola outbreak period and less than a third had ever used any kind of family planning [10].

Various sexually related risk behaviours have been observed among adolescents in African countries. In Ghana, 34% of adolescents aged 10–19 years ever had sex, 73.8% had not used a condom at last sex, and 32.5% had multiple sexual partners [11]; in Namibia in 2004, 33.2% of adolescents aged 10–19 years ever had sex, and 17.1% had multiple sexual partners [12, 13]. Between 2015 and 2017, a community survey of adolescents (15–19 years) in Uganda, Tanzania, Nigeria, Ghana, Eswatini, Ethiopia, and Burkina Faso found that 25.9% had ever had sex. Among sexually active adolescents in this study, the early sexual debut was 21% for girls and 28% for boys, while 46% of girls and 40% of boys had unprotected sex during their last sexual encounter [4].

In a study of 15-year-olds in 30 European countries, Israel, and Canada, 27% had had sexual intercourse, and 14% had not used the contraceptive pill or condoms at their most recent sex [14], and in a study of 15-year-olds in 10 European countries, the prevalence of sexual initiation was 18.8%, and among sexually active, 52.4% had less than one sexual partner [15].

Although the commonness of sexual behaviour varies by country, culture, study methodology and sample size, the relationships between sexual and non-sexual risk behaviours and the function of psychosocial modulators may follow similar patterns. A previous study has identified factors associated with sexual risk behaviour (ever had sex, early sexual debut, no condom use, and no contraceptive use) among adolescents aged 13–16 years old, and they include male sex, older age, substance use, psychological distress, school truancy, and a lack of parental and peer support [16]. Also, substance abuse has been associated with the increased likelihood of youths participating in unsafe sexual practices. When comparing youths who use substances to those who do not, studies show that those who use substances are more likely to engage in early sexual intercourse, have many sexual partners, and use condoms at a lesser rate [17, 18]. Few studies have been undertaken among Liberian youths to investigate the link between risky sexual behaviours and substance abuse. One study revealed no link between alcohol consumption and transactional sex [19], while another identified an association between alcohol consumption and having several sexual partners [7]. Other drugs, such as marijuana, cocaine, and stimulants, such as methamphetamines, have been linked to risky sexual behaviours in adolescence [7, 20, 21].

Gender norms and power dynamics between males and females in Africa are known to promote risky sexual behaviours among adolescents, putting them at risk of sexually transmitted diseases and unplanned pregnancies [22]. Substance abuse has been reported to be associated with gender-based violence among young people, especially among females [23]. Also, inconsistent condom uses among males has been found to be associated with being physically/sexually violent [24]. Gender norms that promote masculinity and expect females not to refuse sexual advances or for not using condoms by their male counterparts make females vulnerable to risky sexual practices [22, 24]. A Liberian study reports that societal norms often blame the female and prioritise protecting family or institution over the safety of the victims when gender-based violence has occurred [25].

Sierra Leone and Liberia are neighbouring countries that have a shared history. Both countries' populations have experienced civil war and, most recently, an Ebola disease outbreak leading to profound economic hardship and psychopathologies such as post-traumatic stress disorder, depression, psychosis, and family disruption [20, 26–28]. These mental health morbidities and family disruption were more profound among adolescents and young people, making them vulnerable to indulging in at-risk sexual behaviours such as early sexual debut, having multiple sexual partners, and not using condoms [10, 29, 30]. Also, the adolescent birth rate in these countries is reported to be high and above the average in sub-Saharan Africa [31]. Given such backgrounds of these two countries, it is important to examine how personal, psychosocial, and protective factors influence sexual risk behaviour, especially among adolescents. Currently, there is limited national data on sexual risk behaviour and related risk factors among only adolescents. Most studies conducted in these countries are either community based or are among adolescents and adults combined [7, 19, 32, 33]. Knowing the prevalence of sexual behaviour and the risk factors associated with it among teenagers in Sierra Leone and Liberia can aid in developing intervention programs aimed at delaying sexual initiation and encouraging “safer sex”. As a result, this study aimed to assess the prevalence and determinants of sexual risk behaviours among school-aged adolescents aged 10-19 years in Sierra Leone and Liberia using the 2017 Sierra Leone and Liberia Global school health survey (GSHS) data.

## Methods

### Sample and procedure

We used publicly available nationally representative cross-sectional datasets of the 2017 Sierra Leone and Liberia Global school health survey [34]. The Sierra Leone and Liberia GSHS employ a two-stage cluster sample design to obtain a nationally representative sample of school-going adolescents aged 10-19 years. The first stage involves the selection of schools with probability proportional to enrolment size, while the second stage involves randomly selecting classes for which all students have equal chances of being selected. In the Sierra Leone GSHS, the school response rate was 94%, the student response rate was 87%, and the overall response rate was 82% [34]. In the Liberia GSHS, the school response rate was 98%, the student response rate was 73%, and the overall response rate was 71% [34]. Our study adheres to STROBE guidelines for observational studies (See supplementary file 1).

## Measures

The questionnaire used in this study and the definition of the variables used in this study is shown in Table 1. The GSHS uses validated core questionnaire modules, core-expanded questions, and country-specific questions that can be self-administered during a normal class period. It consists of validated questions from ten standardised core modules, including nutrition, physical activity, hygiene, mental health, alcohol use, tobacco use, drug use, sexual behaviours, violence/injury, and protective factors [35]. When developing their questionnaire, participating countries are expected to select six of the ten core modules and add any core-expanded questions and country-specific questions about topics unique to their situation. These questions are translated into the appropriate language of instruction and piloted among a sample of students [35]. In the case of Sierra Leone and Liberia, the GSHS questionnaire was administered in English since it is the language of instruction.

**Table 1:** Questionnaire items and coding scheme

Indicator	Item	Responses (coding scheme)
**Outcome Variable (Sexual risk behaviour)**		
Ever sex	‘Have you ever had sexual intercourse?’	‘Yes, No’ (coded yes = 1, no = 0)
Age of sexual initiation	‘How old were you when you had sexual intercourse for the first time?’	‘I have never had sexual intercourse 11 years old or younger to 18 years old or older’
Number of sex partners	‘During your life, with how many people have you had sexual intercourse?’	‘I have never had sexual intercourse, 1 person to 6 or more people’
Condom use	‘The last time you had sexual intercourse, did you or your partner use a condom?’	‘I have never had sexual intercourse, Yes, No, I do not know’
Birth control use	‘The last time you had sexual intercourse, did you or your partner use any method of birth control, such as withdrawal, rhythm (safe time), birth control pills, or any other method to prevent pregnancy?	‘I have never had sexual intercourse, Yes, No, I do not know
**Independent variables**		
**Substance use**		
Current alcohol use	‘During the past 30 days, on how many days did you have at least one drink containing alcohol?’	‘1 = 0 days to 7 = All 30 days (coded 1 = 0, 2–7 = 1)’
Cannabis use	‘During your life, how many times have you used marijuana	‘1 = 0 times to 5 = 20 or more times (coded 1 = 0 and 2–5 = 1)’
Amphetamine use	‘During your life, how many times have you used amphetamines or methamphetamine	‘1 = 0 times to 5 = 20 or more times (coded 1 = 0 and 2–5 = 1)’
**Psychological distress**		
No close friends	‘How many close friends do you have?’	‘1 = 0–4 = 3 or more (coded 1 + = 0, 0 = 1)’
Loneliness	‘During the past 12 months, how often have you felt lonely?’	‘1 = never to 5 = always (coded 1–3 = 0 and 4–5 = 1)’
Anxiety	‘During the past 12 months, how often have you been so worried about something that you could not sleep at night?’	‘1 = never to 5 = always (coded 1–3 = 0 and 4–5 = 1)’
Suicide ideation	‘During the past 12 months, did you ever seriously consider attempting suicide?’	‘Yes, No’
Suicide attempt	‘During the past 12 months, how many times did you actually attempt suicide?’	‘1 = 0 times to 5 = 6 or more times (coded 1 = 0 and 2–5 = 1)’
**Protective factors**		
School attendance	‘During the past 30 days, on how many days did you miss classes or school without permission?’	‘1 = 0 days to 10 or more days (coded 1 = 1)’
Peer support	‘During the past 30 days, how often were most of the students in your school kind and helpful?’	‘1 = never to 5 = always (coded 1–3 = 0 and 4–5 = 1)’
Parental supervision	‘During the past 30 days, how often did your parents or guardians check to see if your homework was done?’	‘1 = never to 5 = always (coded 1–3 = 0 and 4–5 = 1)’
Parental connectedness	‘During the past 30 days, how often did your parents or guardians understand your problems and worries?’	‘1 = never to 5 = always (coded 1–3 = 0 and 4–5 = 1)’
Parental bonding	‘During the past 30 days, how often did your parents or guardians really know what you were doing with your free time?’	‘1 = never to 5 = always (coded 1–3 = 0 and 4–5 = 1)’
Parental respect for privacy	‘During the past 30 days, how often did your parents or guardians go through your things without your approval?’	‘1 = never to 5 = always (coded 1–3 = 0 and 4–5 = 1)’

Sexual risk behaviour was considered as the outcome variable in our study, and it was assessed using the following questions ever having had sexual intercourse, age of sexual debut, number of people who have had sexual intercourse within a lifetime, condom use at last sexual intercourse, and any birth control use other than condom at last sexual intercourse. Sexual risk behaviour was defined as ever having had sex, early sexual debut (< 14 years), having had two or more sexual partners in a lifetime, not using a condom at last sex and no other birth control use at last sex. Composite sexual risk behaviour was defined as having had sex, early sexual debut (< 14 years), having had two or more sexual partners in a lifetime and not using a condom at last sex. As reported in previous studies [36, 37], we excluded no other birth control use because of the overlap with not using a condom at last sex. The Independent variables considered in this study are in Table 1. As in a previous study [36], we considered no close friends, loneliness, anxiety, suicidal ideation, and suicide attempt as psychological distress items. Based on similar study by Pengpid and Pelzer [38], we summed these items into three groups – 0 = 0, 1 = 1 single and 2–5 = 2 multiple. School attendance, peer and parental or guardian support were considered protective factors. The four items that measure parental or guardian support were summed and divided into three groups—0–1 as low, 2 as medium and 3–4 as high support.

### Ethical consideration

No formal ethical approval to conduct this study was necessary, given that our study is based on an analysis of a publicly available deidentified secondary dataset. Notwithstanding, ethics approval was obtained from the Ministries of Health in Sierra Leone and Liberia prior to conducting the surveys in the two countries.

### Data analysis

We analysed our pooled data from the two sets of surveys using SPSS version 27. We employed descriptive statistics to describe our sample. Chi-square statistics were used to compare the independent variables between Sierra Leone and Liberia. Binary regression statistics were used to determine the correlates of individual sexual risk behaviours (non-birth control use at last sex, non-condom use at last sex, multiple sexual partners, early sexual debut, and ever had sex) and a composite measure of multiple sexual risk behaviour. We excluded current tobacco use as an independent variable in our analysis because the data was unavailable in the Sierra Leone GSHS dataset. We employed complex samples analysis in all statistical procedures to account for the sampling weights and the multi-stage design. Statistical significance was set at *p* < 0.05. Hosmer–Lemeshow test was used to test the fitness of the sexual risk behaviour model and it was found to be fit (*p* = 0.277). We tested multicollinearity among explanatory variables using variance inflation factor (VIF). The minimum and maximum VIFs values were 1.017 and 1.422 (See supplementary file 2).

## Results

### Characteristics of the sample and sexual risk behaviour in Sierra Leone and Liberia

Table 2 summarises the characteristics of the sample and sexual risk behaviour in Sierra Leone and Liberia. The sample consisted of 2798 and 2744 school-going adolescents from Sierra Leone and Liberia, respectively. Close to half of them were above the age of 17 years (45.3%) and were females (48.3%). A similar percentage of females was observed in Sierra Leone and Liberia. Overall, close to half of the students have ever had sex (48.4%) with more than a third (38.9%) in Sierra Leone and close to two-thirds (61.9%) in Liberia, *p* =  < 0.001. Among sexually active school-going adolescents, close to a third (31.3%) have had an early sexual debut (< 14 years). However, 44.4% reported early sexual debut in Sierra Leone and less than a quarter (20.6%) in Liberia, *p* =  < 0.001. Close to one in five (19.3%) had multiple sexual partners, 13.0% in Sierra Leone and 27.8% in Liberia, *p* =  < 0.001. The majority (80.2%) of school-going adolescents exhibit multiple sexual risk behaviour, although such risky behaviours were more common among adolescents in Sierra Leone (85.2%) than in Liberia (75.2%), *p* = 0.023.

**Table 2:** Characteristics of the sample and sexual risk behaviour in Sierra Leone and Liberia 2017 GSHS

Study characteristics	Variable	All N (%)	Sierra Leone N (%)	Liberia N (%)	*P*-value
Age in years	14 or less	1365(26.7)	973(35.6)	392(16.1)	< 0.001
	15–16	1452(28.0)	939(34.6)	513(20.2)	
	≥ 17	2624(45.3)	868(29.8)	1756(63.7)	
Sex	Male	2640(51.7)	1258(51.6)	1382(51.8)	0.960
	Female	2737(48.3)	1484(48.4)	1253(48.2)	
**Sexual behaviour**					
Ever had sex	Yes	2181 (48.4)	917(38.9)	1264(61.9)	< 0.001
Early sexual debut (< 14 years)	Yes	536(31.3)	330(44.4)	330(20.6)	< 0.001
Multiple sexual partners	Yes	1044(19.3)	360(13.0)	684(27.8)	< 0.001
No condom use at last sex	Yes	848(48.7)	437(58.6)	411(39.8)	< 0.001
No birth control use at last sex	Yes	892(48.5)	389(50.7)	503(46.6)	0. 424
Multiple sexual risk behaviour	Yes	1142(80.2)	529(85.2)	613(75.3)	0.023
**Substance use**					
Current alcohol use	Yes	1011(18.6)	393(13.4)	618(24.7)	< 0.001
Ever use cannabis	Yes	358(6.6)	165(5.2)	193(8.4)	0.032
Ever use amphetamine use	Yes	371(9.7)	191(7.9)	180(7.9)	0.999
**Psychological distress**					
Have No close friends	Yes	579(10.8)	249(9.2)	330(12.6)	0.003
Felt Lonely	yes	933(16.9)	566(19.4)	367(13.9)	0.014
Anxiety	Yes	1060(19.2)	520(18.5)	540(20.0)	0.339
Suicide ideation	Yes	1076(19.7)	394(14.2)	682(26.3)	< 0.001
Suicide Attempts	Yes	1402(25.7)	541(19.1)	861(33.7)	< 0.001
**Protective factors**					
Missed class/school	Yes	2121(40.5)	992(35.0)	1129(47.6)	< 0.001
Peer support	Yes	1741(32.6)	854(29.6)	887(36.4)	0.015
Parental Support	Low	2153(42.4)	1116(39.6)	1037(46.4)	0.066
	Medium	1348(28.4)	781(30.0)	567(26.1)	
	High	1377(29.2)	771(30.4)	606(27.5)	

### Associations with sexual risk behaviour

Table 3 provides correlates associated with ever had sex among school-going adolescents in Sierra Leone. Adolescents in Liberia were more likely than their Sierra Leonean counterparts to have ever had sex (AOR = 1.548; 95%CI:1.199- 1.999). Among those sexually active adolescents in Liberia, compared to those in Sierra Leone, were more likely to have multiple sexual partners (AOR = 1.587; 95%CI:1.137–2.214). However, adolescents in Liberia were less likely to show multiple sexual risk behaviour than their Sierra Leonean counterparts (AOR = 0.572; 95%CI:0.345–0.946). See Tables 4, 5 and 6.

**Table 3:** Associations with ever had sex among school-going adolescents in Sierra Leone and Liberia 2017 GSHS

**Study characteristics**	**All AOR (95%CI)**	**Sierra Leone** AOR (95%CI)	**Liberia** AOR (95%CI)
Liberia vs Sierra Leone	1.548(1.199- 1.999)		
**Age group**			
14 or less	1	1	1
15–16	1.432(0.967–2.120)	1.554(0.964–2.505)	1.257(0.735–2.149)
≥ 17	**3.469(2.323–5.179)**	**3.086(1.721–5.533)**	**4.048(2.314–7.080)**
**Sex** (Male vs female)	**1.578(1.202- 2.071)**	**1.880(1.280–2.762)**	1.246(0.938–1.656)
**Current alcohol use** (yes vs no)	2.221(1.672–2.948)	1.801(1.063–3.050)	**2.519(1.753–3.621)**
**Ever use cannabis** (Yes vs No)	6.041(2.423–15.060)	5.606(1.792–17.540)	6.436(1.271–32.595)
Ever use amphetamine use (Yes vs No)	1.651(0.851–3.201)	1.594(0.738–3.442)	2.218(0.644–7.639)
**Psychological distress items**			
0	1	1	1
1	**1.586(1.134–2.219)**	**1.883(1.262–2.810)**	**1.273(0.847–1.913)**
2–5	**2.403(1.568–3.683)**	**3.346(1.831–6.117)**	**1.611(1.105–2.350)**
**Missed class/school** (yes vs no)	**1.312(1.084–1.588)**	**1.583(1.136–2.206)**	**1.105(0.822–1.487)**
**Peer support** (Yes vs No)	0.935(0.723–1.209)	0.937(0.629–1.395)	0.920(0.707–1.197)
**Parental support**			
Low	**1.492(1.120–1.987)**	**2.027(1.322–3.107)**	**1.034(0.650–1.644)**
Medium	1.168(0.852–1.602)	1.212(0.797–1.843)	1.115(0.746–1.667)
High	1	1	1

**Table 4:** Associations with Early sexual debut among school-going adolescents in Sierra Leone and Liberia 2017 GSHS

**Study characteristics**	**All AOR (95%CI)**	**Sierra Leone** AOR (95%CI)	**Liberia** AOR (95%CI)
Liberia vs Sierra Leone	0.640(0 .384- 1.067)		
**Age group**			
14 or less	1	1	1
15–16	0.060(0.025–0 .143)	**0.051(0 .016–0 .161)**	**0 .080(0 .025–0 .253)**
≥ 17	0 .021(0 .009–0 .050)	**0 .017(0 .005–0 .057)**	**0 .035(0 .014–0 .086)**
**Sex** (Male vs female)	**1.754(1.151- 2.674)**	1.575(0 .768- 3.231)	**2.513(1.498- 4.215)**
**Current alcohol use** (yes vs no)	1.048(0 .625- 1.757)	1.835(0 .635- 5.301)	0 .635(0 .308- 1.308)
**Ever use cannabis** (Yes vs No)	1.364(0 .566- 3.290)	0.575(0 .192- 1.726)	**7.338(2.004- 26.864)**
Ever use amphetamine use (Yes vs No)	1.475(0 .502- 4.337)	1.199(0 .355- 4.053)	0.987(0.208- 4.695)
**Psychological distress items**			
0	1	1	1
1	1.242(0.718- 2.149)	1.185(0 .589- 2.387)	1.311(0 .743- 2.311)
2–5	**2.217(1.311- 3.748)**	**2.855(1.262- 6.457)**	1.428(0.882- 2.312)
**Missed class/school** (yes vs no)	0 .906(0 .609- 1.347)	0 .922(0 .501- 1.699)	0.877(0 .544- 1.414)
**Peer support** (Yes vs No)	0.879(0.536- 1.441)	1.019(0 .487- 2.132)	0.751(0 .397- 1.422)
**Parental support**			
Low	1.183(0 .782 1.790)	1.071(0 .512- 2.242)	1.310(0 .669- 2.565)
Medium	1.427(0 .814- 2.501)	1.621(0 .723- 3.633)	1.359(0 .533- 3.469)
High	1	1

**Table 5:** Associations with multiple sexual partners among school-going adolescents in Sierra Leone and Liberia 2017 GSHS

**Study characteristics**	**All AOR (95%CI)**	**Sierra Leone** AOR (95%CI)	**Liberia** AOR (95%CI)
Liberia vs Sierra Leone	**1.587(1.137–2.214)**		
**Age group**			
14 or less	1	1	1
15–16	1.686(1.105–2.574)	**1.666(1.013–2.739)**	**1.778(0.800–3.949)**
≥ 17	**4.463(2.890–6.894)**	**3.288(1.974–5.476)**	**6.648(3.130–14.121)**
**Sex** (Male vs female)	**2.232(1.553–3.208)**	**2.542(1.551–4.166)**	**1.992(1.294–3.066)**
**Current alcohol use** (yes vs no)	**2.981(2.048–4.340)**	**3.219(1.563–6.630)**	**2.626(1.886–3.655)**
**Ever use cannabis** (Yes vs No)	1.812(0.904–3.632)	**2.372(1.095–5.136)**	1.316(0.542–3.197)
Ever use amphetamine use (Yes vs No)	1.257(0.695-.2.273)	1.477(0.657–3.318)	0.785(0.335–1.836)
**Psychological distress items**			
0	1	1	1
1	1.245(0.810–1.915)	1.179(0.575–2.420)	1.262(0.764–2.087)
2–5	1.403(0.955–2.061)	1.324(0.650–2.697)	1.431(0.998–2.053)
**Missed class/school** (yes vs no)	**1.683(1.338–2.118)**	**1.854(1.304–2.637)**	**1.582(1.145–2.187)**
**Peer support** (Yes vs No)	1.146(0.889–1.477)	1.423(0.973–2.080)	0.965(0.710–1.312)
**Parental support**			
Low	1.178(0.845–1.640)	1.768(0.925–3.378)	0.859(0.604–1.221)
Medium	0.972(0.669–1.413)	1.098(0.658–1.834)	0.940(0.581–1.523)
High	1	1

**Table 6:** Associations with multiple sexual risk behaviour among school-going adolescents in Sierra Leone and Liberia 2017 GSHS

**Study characteristics**	**All AOR (95%CI)**	**Sierra Leone** AOR (95%CI)	**Liberia** AOR (95%CI)
Liberia vs Sierra Leone	**0.572(0.345–0.946)**		
**Age group**			
14 or less	1	1	1
15–16	0.305(0.089–1.046)	0.200(0.0340–1.156)	0.663(0.217–2.023)
≥ 17	0.188(0.063–0.558)	0.091(0.017–0.483)	0.650(0.224–1.883)
**Sex** (Male vs female)	**2.310(1.543–3.458)**	**3.632(1.852–7.122)**	**1.913(1.156–3.167)**
**Current alcohol use** (yes vs no)	**3.064(2.137–4.392)**	29.012(2.303–365.529)	**1.923(1.190–3.107)**
**Ever use cannabis** (Yes vs No)	0.278(0.073–1.056)	0.100(0.025–0.403)	2.123(0.445–10.134)
Ever use amphetamine use (Yes vs No)	1.739(0.444–6.801)	0.863(0.159–4.678)	0.959(0.172–5.338)
**Psychological distress items**			
0	1	1	1
1	1.188(0.745–1.894)	1.459(0.468–4.554)	1.129(0.644–1.982)
2–5	1.324(0.805–2.177)	1.451(0.507–4.148)	1.132(0.683–1.875)
**Missed class/school** (yes vs no)	**1.655(1.133–2.418)**	1.213(0.614–2.399)	**1.921(1.289–2.865)**
**Peer support** (Yes vs No)	1.350(0.799–2.280)	1.179(0.482–2.883)	1.238(0.687–2.231)
**Parental support**			
Low	1.322(0.898–1.946)	1.611(0.608–4.270)	1.131(0.728–1.758)
Medium	1.132(0.754–1.698)	1.100(0.440–2.746)	1.249(0.746–2.094)
High	1	1	1

Regarding respondent's age, adolescents who were 17 years and older were more likely than those 14 years and younger to have ever had sex (AOR = 3.469; 95% CI: 2.323–5.179). A similar relationship was observed among adolescents in Sierra Leone (AOR = 3.086; 95%CI:1.721–5.533) and Liberia (AOR = 4.048; 95%CI:2.314–7.080). Also, among sexually active adolescents, a similar association was observed between age and not using other birth control methods other than condoms (AOR = 2.474; 95%CI:1.359–4.505). Regarding sex, overall, male adolescents were more likely than their female counterparts to have ever had sex (AOR = 1.578; 95%CI:1.202- 2.071), to have had an early sexual debut (AOR = 1.754;95%CI:1.151–2.674) had multiple sexual partners (AOR = 2.232; 95%CI:1.553–3.208), multiple sexual risk behaviour (AOR = 2.310;95%CI:1.543–3.458). Males were associated with ever had sex, and such association was observed among Sierra Leoneans (AOR = 1.880; 95%CI:1.280–2.762) but not among Liberian adolescents (AOR = 1.246; 95%CI:0.938–1.656). Also, among sexually active adolescents, males were more likely than females to not use other birth control methods other than condom in Sierra Leone (AOR = 1.827; 95%CI:1.063–3.141) but not in Liberia (AOR = 0.814; 95%CI:0.559–1.186). However, being male gender was associated with high-risk sexual behaviour in Sierra Leone (AOR = 3.632; 95%CI:1.852–7.122) and Liberia (AOR = 1.913; 95%CI:1.156–3.167). Adolescents who were current alcohol users were more likely than non-users to have ever had sex (AOR = 2.221;95%CI:1.672–2.948), had multiple sexual partners (AOR = 2.981; 95%CI:2.048–4.340) and had high-risk sexual behaviour (AOR = 3.064; 95%CI:2.137–4.392). See Tables 3, 5, 6 and 7.

**Table 7:** Associations with No birth control use other than condom among school-going adolescents in Sierra Leone and Liberia 2017 GSHS

**Study characteristics**	**All AOR (95%CI)**	**Sierra Leone** AOR (95%CI)	**Liberia** AOR (95%CI)
Liberia vs Sierra Leone	1.074(0.665–1.735)		
**Age group**			
14 or less	1	1	1
15–16	1.660(0.898–3.069)	1.915(0.839–4.374)	1.312(0.512–3.365)
≥ 17	**2.474(1.359–4.505)**	**3.273(1.430–7.492)**	1.851(0.973–3.518)
**Sex** (Male vs female)	1.181(0.841–1.659)	**1.827(1.063–3.141)**	0.814(0.559–1.186)
**Current alcohol use** (yes vs no)	0.750(0.499–1.126)	0.331(0.098–1.122)	1.149(0.878–1.503)
**Ever use cannabis** (Yes vs No)	1.544(0.733–3.253)	3.023(0.949–9.632)	0.663(0.255–1.723)
Ever use amphetamine use (Yes vs No)	1.007(0.482–2.103)	1.326(0.508–3.460)	1.062(0.263–4.288)
**Psychological distress items**			
0	1	1	1
1	1.071(0.547–2.097)	1.357(0.472–3.901)	0.898(0.567–1.423)
2–5	1.283(0.730–2.256)	1.877(0.622–5.666)	1.131(0.716–1.786)
**Missed class/school** (yes vs no)	0.966(0.733–1.273)	1.146(0.698–1.881)	0.896(0.642–1.250
**Peer support** (Yes vs No)	0.868(0.683–1.103)	**0.577(0.342–0.975)**	1.139(0.809–1.603)
**Parental support**			
Low	**0.697(0.498- 0.977)**	0.510(0.250–1.042)	0.826(0.571–1.195)
Medium	1.102(0.704–1.726)	0.784(0.471–1.307)	1.188(0.723–1.950)
High	1	1	1

Overall, adolescents with one (AOR = 1.586; 95%CI:1.134–2.219) and two or more (AOR = 2.403; 95%CI:1.568–3.683) forms of psychological distress were more likely to have ever had sex than those who do not show any form of psychological distress with a similar pattern seen in Sierra Leone and Liberia. Also, adolescents that showed two or more forms of psychological distress were more likely to have had an early sexual debut (AOR = 2.217; 95%CI:1.311- 3.748), with a similar relationship observed only among Sierra Leonean adolescents (AOR = 2.855; 95%CI:1.262- 6.457). In addition, adolescents that exhibited one form of psychological distress were more likely not to use a condom in their last sexual intercourse (AOR = 1.346; 95%CI:1.016–1.783). See Tables 3, 4 and 8.

**Table 8:** Associations with no condom use among school-going adolescents in Sierra Leone and Liberia 2017 GSHS

**Study characteristics**	**All AOR (95%CI)**	**Sierra Leone** AOR (95%CI)	**Liberia** AOR (95%CI)
Liberia vs Sierra Leone	0.614(0.362–1.041)		
**Age group**			
14 or less	1	1	1
15–16	0.919(0.524–1.613)	0.943(0.398–2.234)	0.732(0.344–1.555)
≥ 17	0.686(0.398–1.181)	0.722(0.351–1.485	0.535(0.250–1.143)
**Sex** (Male vs female)	1.174(0.733–1.882**)**	1.060(0.502–2.242)	1.209(0.810–1.806)
**Current alcohol use** (yes vs no)	1.106(0.767–1.596)	0.954(0.366–2.485)	1.075(0.765–1.509)
**Ever use cannabis** (Yes vs No)	0.622(0.193–1.997)	0.418(0.093–1.877)	1.533(0.819–2.868)
Ever use amphetamine use (Yes vs No)	1.755(0.693–4.446)	2.544(0.634–10.206)	0.754(0.295–1.929)
**Psychological distress items**			
0	1	1	1
1	**1.346(1.016–1.783)**	1.775(0.961–3.282)	1.138(0.812–1.595)
2–5	0.772(0.505–1.180)	0.800(0.352–1.817)	0.809(0.522–1.254)
**Missed class/school** (yes vs no)	1.226(0.874–1.718)	1.244(0.747–2.074)	1.174(0.834–1.652)
**Peer support** (Yes vs No)	0.784(0.514–1.196)	1.019(0.499–2.080)	**0.608(0.435–0.850)**
**Parental support**			
Low	1.183(0.792–1.767)	1.565(0.778–3.151)	0.979(0.646–1.482)
Medium	0.885(0.605–1.294)	0.813(0.425–1.558)	1.003(0.637–1.580)
High	1	1	1

Adolescents who missed school were more likely to have had sex (AOR = 1.312;95%CI:1.084–1.588) and had multiple sexual partners (AOR = 1.683; 95%CI:1.338–2.118) and multiple sexual risk behaviour (AOR = 1.655; 95%CI:1.133–2.418). Peer support was not associated with any of the sexual risk indicators. Adolescents with less parental support were more likely to have ever had sex (AOR = 1.492; 95%CI:1.120–1.987). A similar relationship was seen among adolescents in Sierra Leone (AOR = 2.027; 95%CI:1.322–3.107) but not in Liberia (AOR = 1.034; 95%CI: 0.650–1.644). However, adolescents with less parental support were less likely to show multiple sexual behaviours (AOR = 0.697; 95%CI:0.498- 0.977). See Tables 3, 5, and 6.

## Discussion

Our study found a high prevalence of sexual risk behaviours (ever had sex, early sexual debut, multiple sexual partners, no condom, and no birth control use at last sex) among school-going adolescents, with Liberian adolescents showing lesser odds of indulging in multiple sexual risk behaviours than their Sierra Leonean counterparts. Close to half of them have ever had sex (48.4%), with high prevalence observed among Liberians (61.9%) than Sierra Leoneans (38.2%) school-going adolescents. Our finding for both countries is lower than what was reported among school-going adolescents aged (11-18 years in Mozambique and aged 14–19 years in Ethiopia [36, 39] but higher than what was reported in school-going adolescents aged (10-19 years) in Ghana [11] and in four Caribbean countries [40] and five East Asia countries [41]. However, in individual countries, the reported prevalence in Mozambique was higher than what is found for Sierra Leone but low regarding our finding for Liberia.

Close to a third in both countries had an early sexual debut (< 14 years), with a higher prevalence seen among Sierra Leonean school-going adolescents, and this was lower than what other similar studies reported in the Caribbean and Asian countries [40, 41]. However, the prevalence of early sexual debut in Sierra Leone and Liberia was consistent and higher than reported in studies conducted in Ethiopia and Mozambique [36, 39], respectively. In addition, close to half (48.7%) did not use a condom in their last sex, with a higher prevalence seen among Sierra Leoneans (58.6%) than Liberians (39.8%), and our findings for both countries are higher than the prevalence reported in studies conducted in Mozambique, Ghana and Ethiopia [11, 36, 39]. Our study's high prevalence of risky sexual behaviour is consistent with previous community-based studies conducted among adolescents and youths in both countries [7–9, 33] and similar studies in other African countries [1, 4, 11, 36]. The higher prevalence of risky sexual behaviour in our study may be attributed to increased trauma and economic hardships, changes in parenting styles, breakdown in the social fabric in our society and broken homes experienced by young people, which is due to civil war and lately the Ebola outbreak in these two countries [42–44]. Risky sexual behaviour, such as inconsistent condom use by male adolescents, has been linked with physical and or sexual violence against their female counterparts [22, 24]. Such violence is known to have been promoted by social-cultural norms such as male dominance over women exacerbated by the civil wars and Ebola outbreaks these countries had witnessed [45]. Based violence limits adolescent girls’ and women’s decision-making power regarding their reproductive health, making them vulnerable to sexually transmitted diseases and teenage pregnancy [46].

Consistent with previous studies conducted in Ghana [11], Mozambique [36], Fiji, Kiribati, Samoa, and Vanuatu [16], among the same age range as in our study, males were more likely to have ever had sex, had an early sexual debut (< 14 years), multiple sexual partners and high sexual risk behaviour composite score. Similarly, being 17 years and older was associated with being sexually active, having multiple sexual partners, and non-birth control uses other than condoms. Our findings suggest the need to develop and implement gender and age-specific interventions to help prevent adolescents from indulging in risky sexual behaviours. Substance use (alcohol and cannabis use) was associated with sexual risk behaviour among adolescents in Sierra Leone and Liberia. Our finding aligns with previous studies conducted in Liberia [7] and some African countries [11, 36]. Substance abuse has been reported to be higher among adolescent in Sierra Leone and Liberia [20, 21, 47], and such behaviour have been reported to be associated with risky sexual behaviour and teenage pregnancy [7, 48]. Substance abuse has been found to be associated with gender-based violence, especially among female adolescents. Female adolescents are prone to gender-based violence because of social norms promote male dominance and power imbalance in favour of males regarding sexual and reproductive health decision-making. Also, societal norms do not protect females as victims of gender-based violence. These include putting the blame on victims rather than the perpetrator, protecting family or institution rather than the rights and safety of the victim [25].

In contrast to a Mozambican study [36] but consistent with a Ghanaian study [11] and in the Caribbean as well as Asian pacific island countries [16, 40, 41], psychological distress was associated with sexual risk behaviours such as ever had sex, early sexual debut and non-condom use. High mental health burden has been reported among adolescents in Sierra Leone and Liberia, and such burden is linked to exposure to trauma during the civil war and Ebola outbreak in these two countries [26, 30]. Psychological distress contributes to adolescents’ vulnerability, leading to risky sexual behaviour, violence, and substance abuse, partly caused by economic hardship and parental loss [49]. Although peer support was identified as a protective factor in only one of the sexual risk behaviour indicators (non-condom use) among Liberian adolescents, previous studies have concluded that negative peer influence affects adolescents' sexual risk behaviour [27, 50]. Low parental support was associated as with ever had sex but was not linked with the other sexual risk behaviour indicators, which is consistent with other studies in which parental support was a protective factor for non-condom use and non-birth control use at last sex but not multiple sexual risk behaviours [11, 36]. Our finding may reflect the significant trauma due to war, poverty and infectious disease outbreaks in these countries leading to mental health needs that have not been fully addressed. Such an unmet need makes it difficult for adolescents to benefit from protective factors such as peer and parental support. The association between less parental support and ever had sex was significant among adolescents in Sierra Leone but not in Liberia. It is possible that parents in Liberia are more involved in their children life compared to Sierra Leonean parents, and that might explain our finding. However, further study needs to be done in this area to confirm or discard our speculation.

### Policy and practice implication

Our findings underscored the need for strengthening sexual and reproductive health education in schools and communities. Such educational programs should integrate mental health promotion activities targeting adolescent-specific health needs, such as increasing their access to counselling and education. Also, peer support and parental involvement in adolescent daily activities will help reduce adolescent sexual risk behaviour.

### Study limitations

The 2017 Sierra Leone and Liberia GSHS employed a cross-sectional study design, and as such, we cannot infer causal relationships between our dependent and independent variables. Also, our findings only apply to school-going adolescents in these two countries. Future research should focus on both in-school and out-of-school adolescents. There is a tendency for recall bias as responses were based on self-report.

## Conclusion

Our study suggests that most school-going adolescents in Liberia and Sierra Leone have indulged in multiple sexual risk behaviours, although those in Liberia had fewer odds of being involved in multiple sexual risk behaviours than their Sierra Leone counterparts. Sex, substance use, psychological distress and missing classes were associated with multiple sexual risk behaviours. Peer and parental support were the only protective factors for no condom use among Liberian adolescents and being sexually active among Sierra Leonean adolescents. Our finding highlights the need to strengthen sexual and reproductive health education in schools and communities that incorporate mental health promotion activities.

## Supplementary Information

**Additional file 1.** STROBE Statement—Checklist of items that shouldbe included in reports of cross-sectional studies.  

**Additional file 2.** Test for multicollinearity.

## Data Availability

The dataset informing the findings of this study is publicly available. It can be freely accessed via the WHO NCD Microdata Repository. https://extranet.who.int/ncdsmicrodata/index.php/catalog/GSHS.

## Abbreviations

CI: Confidential Intervals
GSHS: Global School Health Survey
OR: Odds ratio
SPSS: Statistical Package for The Social Sciences
