# Supplementary material for: Sexual risk behaviour among school-going adolescents in Sierra Leone and Liberia: a secondary analysis of the 2017 Global school-based student health surveys
Source: Contracept Reprod Med. 2022 Dec 24;7:27. doi: 10.1186/s40834-022-00193-w (PMC9790129; doi:10.1186/s40834-022-00193-w)
Supplement: Supplementary file 1 — Additional file 1. STROBE Statement—Checklist of items that shouldbe included in reports of cross-sectional studies. [file 40834_2022_193_MOESM1_ESM.doc]

STROBE Statement—Checklist of items that should be included in reports of ***cross-sectional studies***

|  | Item No | Recommendation |
| --- | --- | --- |
| **Title and abstract** | 1 | (*a*) Indicate the study’s design with a commonly used term in the title or the abstract |
|  |  | (*b*) Provide in the abstract an informative and balanced summary of what was done and what was found. See Pages 1-3 |
| Introduction | | |
| Background/rationale | 2 | Explain the scientific background and rationale for the investigation being reported. See pages 4-6 |
| Objectives | 3 | State specific objectives, including any prespecified hypotheses See page 6 |
| Methods | | |
| Study design | 4 | Present key elements of study design early in the paper See page 7 |
| Setting | 5 | Describe the setting, locations, and relevant dates, including periods of recruitment, exposure, follow-up, and data collection. See pages 6-7 |
| Participants | 6 | (*a*) Give the eligibility criteria, and the sources and methods of selection of participants See page 6 |
| Variables | 7 | Clearly define all outcomes, exposures, predictors, potential confounders, and effect modifiers. Give diagnostic criteria, if applicable. See pages7-8 |
| Data sources/ measurement | 8* | For each variable of interest, give sources of data and details of methods of assessment (measurement). Describe comparability of assessment methods if there is more than one group. See pages7-8 |
| Bias | 9 | Describe any efforts to address potential sources of bias. See page 8 |
| Study size | 10 | Explain how the study size was arrived at. See page 7 |
| Quantitative variables | 11 | Explain how quantitative variables were handled in the analyses. If applicable, describe which groupings were chosen and why. See page 8 |
| Statistical methods | 12 | (*a*) Describe all statistical methods, including those used to control for confounding. See page 8 |
|  |  | (*b*) Describe any methods used to examine subgroups and interactions. See page 8 |
|  |  | (*c*) Explain how missing data were addressed. See page 8 |
|  |  | (*d*) If applicable, describe analytical methods taking account of sampling strategy. See page 8 |
|  |  | (*e*) Describe any sensitivity analyses. Not applicable |
| Results | | |
| Participants | 13* | (a) Report numbers of individuals at each stage of study—eg numbers potentially eligible, examined for eligibility, confirmed eligible, included in the study, completing follow-up, and analysed. See page 9 |
|  |  | (b) Give reasons for non-participation at each stage. Not applicable |
|  |  | (c) Consider use of a flow diagram. Not applicable |
| Descriptive data | 14* | (a) Give characteristics of study participants (eg demographic, clinical, social) and information on exposures and potential confounders. See page 9 |
|  |  | (b) Indicate number of participants with missing data for each variable of interest |
| Outcome data | 15* | Report numbers of outcome events or summary measures. . See page 9-10 |
| Main results | 16 | (*a*) Give unadjusted estimates and, if applicable, confounder-adjusted estimates and their precision (eg, 95% confidence interval). Make clear which confounders were adjusted for and why they were included. . See pages 9-11 |
|  |  | (*b*) Report category boundaries when continuous variables were categorized. Not applicable |
|  |  | (*c*) If relevant, consider translating estimates of relative risk into absolute risk for a meaningful time period. See pages 9-11 |
| Other analyses | 17 | Report other analyses done—eg analyses of subgroups and interactions, and sensitivity analyses. See pages 9-11 |
| Discussion | | |
| Key results | 18 | Summarise key results with reference to study objectives. Seevpages 11-12 |
| Limitations | 19 | Discuss limitations of the study, taking into account sources of potential bias or imprecision. Discuss both direction and magnitude of any potential bias.. See page 13 |
| Interpretation | 20 | Give a cautious overall interpretation of results considering objectives, limitations, multiplicity of analyses, results from similar studies, and other relevant evidence. See page 13 |
| Generalisability | 21 | Discuss the generalisability (external validity) of the study results. See page 13 |
| Other information | | |
| Funding | 22 | Give the source of funding and the role of the funders for the present study and, if applicable, for the original study on which the present article is based. See page 14 |

*Give information separately for exposed and unexposed groups.

**Note:** An Explanation and Elaboration article discusses each checklist item and gives methodological background and published examples of transparent reporting. The STROBE checklist is best used in conjunction with this article (freely available on the Web sites of PLoS Medicine at http://www.plosmedicine.org/, Annals of Internal Medicine at http://www.annals.org/, and Epidemiology at http://www.epidem.com/). Information on the STROBE Initiative is available at www.strobe-statement.org.
