# Supplementary material for: Sexual risk behaviour among school-going adolescents in Sierra Leone and Liberia: a secondary analysis of the 2017 Global school-based student health surveys
Source: Contracept Reprod Med. 2022 Dec 24;7:27. doi: 10.1186/s40834-022-00193-w (PMC9790129; doi:10.1186/s40834-022-00193-w)
Supplement: Supplementary file 2 — Additional file 2. Test for multicollinearity. [file 40834_2022_193_MOESM2_ESM.docx]

**Supplementary file 2:** **Test for multicollinearity**

**Multicollinearity diagnosis for the sexual risk behaviour model**

| Independent variables | Tolerance | VIF |
| --- | --- | --- |
| Sex | 0.980 | 1.021 |
| Age | 0.799 | 1.251 |
| Current alcohol use | 0.897 | 1.114 |
| Ever Cannabis use | 0.730 | 1.369 |
| Ever Amphetamine use | 0.703 | 1.422 |
| Psychological distress items | 0.983 | 1.017 |
| School attendance | 0.918 | 1.090 |
| Peer support | 0.899 | 1.112 |
| Parental Support | 0.874 | 1.144 |
| Country | 0.750 | 1.334 |
| Mean |  |  |
